# Strengthening Healthcare Capacity Through a Responsive, Country-Specific, Training Standard: The KITSO AIDS Training Program’s Sup-port of Botswana’s National Antiretroviral Therapy Rollout

**DOI:** 10.2174/1874613600802010010

**Published:** 2008-02-29

**Authors:** Christine Bussmann, Philip Rotz, Ndwapi Ndwapi, Daniel Baxter, Hermann Bussmann, C. William Wester, Patricia Ncube, Ava Avalos, Madisa Mine, Elang Mabe, Patricia Burns, Peter Cardiello, Joseph Makhema, Richard Marlink

**Affiliations:** Harvard School of Public Health, 651 Huntington Ave., Boston, MA 02115, USA

## Abstract

In parallel with the rollout of Botswana’s national antiretroviral therapy (ART) program, the Botswana Ministry of Health established the KITSO AIDS Training Program by entering into long-term partnerships with the Botswana–Harvard AIDS Institute Partnership for HIV Research and Education and others to provide standardized, country-specific training in HIV/AIDS care. The KITSO training model has strengthened human capacity within Botswana’s health sector and been indispensable to successful ART rollout. Through core and advanced training courses and clinical mentoring, different cadres of health care workers have been trained to provide high-quality HIV/AIDS care at all ART sites in the country. Continuous and standardized clinical education will be crucial to sustain the present level of care and successfully address future treatment challenges.

## INTRODUCTION

Over the last five years unprecedented efforts have been made to extend the benefits of antiretroviral therapy (ART) to sub-Saharan Africa, the region of the world hardest hit by the HIV/AIDS pandemic. Botswana, a southern African country of 1.7 million people with one of the highest rates of HIV infection in the world, introduced a national ART program in January 2002. Thirty-two country-wide treatment sites were established by the close of 2004, and over 80,000 persons currently receive treatment [1].

The lack of trained medical personnel has been a major obstacle to the timely and effective rollout of ART programs in Africa [2-6]. Unlike resource-rich countries with low HIV prevalence, where HIV/AIDS care is often provided by specialists, most African nations must marshal their entire healthcare sector to confront the pandemic [7]. The expedited and targeted training of a critical mass of health care workers (HCWs), followed by more broad-based workforce education, is of paramount importance to the success of ART programs in particular and to healthcare sectors in general [8-10].

The KITSO AIDS Training Program, also known simply as KITSO, was created in 2001 by the Botswana Ministry of Health (MOH) and the Botswana–Harvard School of Public Health AIDS Initiative Partnership for HIV Research and Education (BHP), with financial support from the African Comprehensive HIV/AIDS Partnerships (ACHAP), a collaboration between the Government of Botswana, the Bill and Melinda Gates Foundation, and the Merck Company Foundation/Merck &amp; Co. Inc. In 2004, the MOH expanded

KITSO to incorporate additional training partners in the country.

As a training program, KITSO has a number of distinguishing characteristics: (**1**) the program is Botswana’s national training program directed by the MOH; (**2**) the program has a country-specific curriculum, developed in Botswana for practitioners and patients in Botswana; (**3**) the program’s training modules are standardized and have been implemented in a consistent fashion to train a high volume of health staff; (**4**) the program has been responsive to the evolving training needs of Botswana’s health sector since its inception in 2001.

According to the Botswana MOH, KITSO has been instrumental in Botswana’s ART rollout. We report herein the design and implementation of the program, along with its essential principles of operation.

## METHOD

### Country-Specific National Program

With the help of a private donation and the partnership of the MOH and BHP, the initial KITSO AIDS Training Program was developed in 2001 after a country-wide needs assessment. In 2002, Botswana announced the provision of free ART to all eligible citizens according to national treatment guidelines. Without the benefit of a medical school in-country, the MOH partnered with BHP and ACHAP to devise and implement a standardized program to train Botswana’s HCWs in HIV/AIDS care and ART. Following the needs assessment and extensive stakeholder consultations, a national faculty, supported by international advisers, designed country-specific training courses in HIV/AIDS care and treatment, establishing the KITSO AIDS Training Program. KITSO is the Setswana word for “knowledge” and also serves as an acronym for “Knowledge Innovation and Training Shall Overcome” AIDS.

### Standardized Curricula

**Fig. (1):**
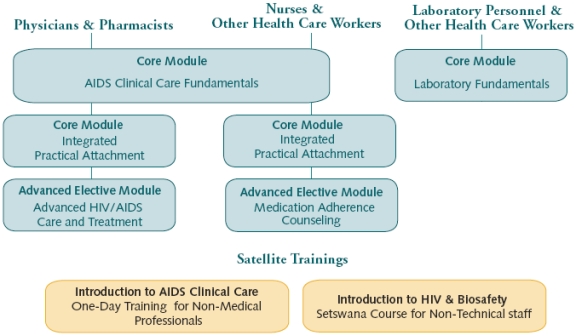
KITSO-BHP curriculum delivery diagram.

The KITSO training modules serve as the standard for training in HIV/AIDS care and treatment throughout Botswana’s public and private healthcare systems. KITSO consists of four didactic and practical core modules to facilitate the ART rollout, two advanced modules to consolidate and extend the HIV/AIDS knowledge and experience of Botswana’s HCWs, and two satellite modules for non-medical staff (Fig. **1**).

Module implementation is standardized for all KITSO courses. Full attendance is required, and participants complete both a baseline assessment and a final examination. HCWs who meet the requirements set by the MOH receive certificates verifying successful course completion.

Regular monitoring and evaluation has been performed since the program’s inception to assess the quality, integrity, and standardization of the training program.

### Core Modules

*AIDS Clinical Care Fundamentals (ACCF),* a three-day didactic training course for doctors, pharmacists, nurses, pharmacy technicians, and social workers was designed to prepare Botswana’s HCWs with the information and skills necessary to begin provide basic ART. This baseline module, which includes both adult and pediatric content, is comprised of lectures, case study discussions, practice exercises, and question and answer sessions. During the course, the following 12 core topic areas are addressed: Introduction to the Botswana National ARV Program, HIV Pathophysiology and Epidemiology, Laboratory Diagnostics in ARV Therapy, Principles of ARV Therapy, Pediatric Considerations and ARV Dosing, ARV Drug Toxicity, Drug-Drug Interactions, ARV Drug Resistance and Treatment Failure, Adherence, Adult and Pediatric Opportunistic Infections, Prevention of Mother-to Child Transmission (PMTCT), and Post-Exposure Prophylaxis.

*Integrated Practical Attachment*****was designed as a hands-on follow-up to *ACCF*, utilizing the high-volume ART clinics established at Botswana’s two referral hospitals for clinical mentoring in adult and pediatric ART. Doctors, nurses, and pharmacy staff are taught under the guidance of experienced national clinicians, who in turn are supported by international experts from various MOH collaborators. Doctors attach for four weeks of mentorship, maintained a clinical logbook, and gained the confidence and ability to see patients independently, while working under expert supervision and support. Nurses and pharmacy staff attach to the same ART clinics for two week rotations to gain experience in the national protocols for adherence counseling, treatment initiation, and treatment follow-up.

*Laboratory Fundamentals*, a one-day didactic and practical module, was designed for laboratory personnel and HCWs involved in the collection and processing of laboratory specimens for the ART program. This training includes practical background on the testing protocols for the ART program, sample collection, labeling, testing procedures, results interpretation and reporting, and basic biosafety.

### Advanced Modules

*Advanced HIV/AIDS Care and Treatment* is an intense five-day course for doctors and pharmacists who have successfully completed *ACCF* and have subsequently gained clinical experience in ART. The course addresses advanced ARV treatment challenges and provides in-depth information in the areas of HIV virology and immunology, ARV drug resistance, principles of ARV therapy, management of treatment failure, management of opportunistic infections, PMTCT, pediatric-specific issues, and relevant findings from HIV/AIDS research conducted in Botswana and throughout the world. This didactic module includes extensive case study discussions, incorporating actual patient cases provided by course participants, to illustrate and reinforce****optimal care and treatment within the Botswana setting.

The *Medication Adherence Counseling (MAC)* module is a three-day interactive training module for nurses, pharmacy technicians, and social workers from active ARV treatment centers. Focusing on potential barriers to medication adherence in the Botswana context, this course combines lectures, case discussions, role-playing, and interactive activities to strengthen adherence counseling skills.

### Satellite Modules

Two one-day satellite training modules—*Introduction to HIV and Biosafety, *a course taught in Setswana for support staff (orderlies, cleaners, drivers, etc.) and *Introduction to AIDS Clinical Care* for lay counselors, family welfare educators, and other non-medical professionals—provide non-medical staff with training relevant to their responsibilities.

### Responsive and Flexible Implementation

The model of training for HCWs in Botswana has mirrored the MOH’s general approach for the ART program. Training was initially a central, vertical effort to equip core treatment teams to participate in the ART rollout. Following this, training courses have been implemented as ongoing ART site support. This expansion involved the introduction of two advanced modules and continued training in *AIDS Clinical Care Fundamentals* targeting HCWs recruited from all facilities in response to the continued decentralization of the national ART program.

KITSO courses, initially implemented centrally in the capital, are now implemented in centralized, regional, and facility-based formats.

#### ART Rollout Training

Botswana’s rollout of ART to 32 treatment sites countrywide was accomplished through a step-wise approach. The initial 32 sites included a main clinic, usually termed an IDCC (Infectious Disease Care Clinic) which was integrated into the existing hospital and linked to 2-5 designated satellite clinics that conduct screening and referrals. Treatment sites were first established in areas of high population density and easy accessibility, followed by the rollout of ART to smaller and more rural sites.

The training preparation of a critical mass of HCWs at treatment sites was an essential step for the successful countrywide rollout of ART [2]. Core Treatment Teams, which were prioritized for training, consisted of 20 staff from the hospital IDCC and four staff from each satellite clinic. The hospital team was commonly comprised of 2-4 doctors, 10-12 nurses, 2-4 pharmacy staff, and 1-2 social workers. The following approach was adopted to deliver didactic training and clinical mentoring for each site:

(**1**) Core Treatment Teams first received training in *AIDS Clinical Care Fundamentals* in either a facility-based or centralized format, depending on the size of the treatment site. Large treatment sites able to release sizable groups of HCWs for training were offered facility-based training, while smaller treatment sites with staff limitations were pooled with other facilities of similar size, to receive centralized training over a period of four to six weeks (e.g., releasing five staff per week over four weeks to accomplish the training of 20 staff). The training format for smaller facilities enabled simultaneous training of Core Treatment Teams from multiple sites, while avoiding the interruption of ongoing health care services which would have resulted from taking all HCWs from a site for training at one time.

(**2**) After the successful completion of *ACCF* training, one doctor, one nurse, and one pharmacy staff from each treatment site were attached to one of the two referral hospitals for the *Integrated Practical Attachment*. At the conclusion of the attachment training, participants returned to their treatment sites equipped to begin ARV efforts there and to provide basic mentorship to other HCWs at that facility.

(**3**) As each treatment site’s opening date approached, the *Laboratory Fundamentals *module was conducted at the facility. For support staff, the satellite module, *Introduction to HIV &amp; Biosafety*, was implemented alongside this training, usually on consecutive days.

(**4**) Before the launch of each treatment site, an experienced ART practitioner (preceptor) from the ACHAP Clinical Preceptorship Program was assigned to the treatment site for 3-6 months, to assist in the final preparations and the initial ART launch, providing ongoing logistical assistance, clinical mentoring, and lectures [6, 11].

#### Site Support Training

Following the country-wide rollout of ART, KITSO introduced *Medication Adherence Counseling* and *Advanced HIV/AIDS Care and Treatment* to build on prior training and hands-on experience among ART providers. Both courses were developed to provide responsibility-specific continuing and advanced training support for HCWs from treatment sites providing ART. These courses, conducted in a centralized format, build specialized knowledge and skills integral to the long-term success of Botswana’s ART program.

These courses—and the *Introduction to AIDS Clinical Care* training for family welfare educators, lay counselors, and health educators—strengthen the ART program and improve the quality of care received by patients in Botswana. *Introduction to AIDS Clinical Care* is conducted at ART sites by the BHP-PEPFAR Master Trainer Program, another MOH-coordinated training program that was begun more recently to provide on-site supportive training, mentoring, and clinic management assistance to operating ART sites.

In addition, following the initial training of core HCW teams for each treatment site, *ACCF* is now being provided to all HCWs in Botswana. Staff reassignment, resignation, and replacement create a continuing need for the baseline training provided in the *ACCF* module.

## RESULTS

**Fig. (2):**
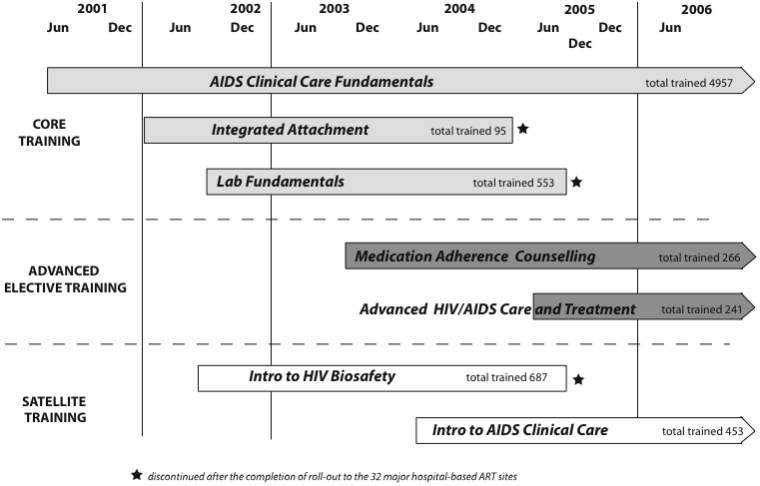
Training course implementation: time line and training output.

KITSO training has been integral for the skills preparation of all 32 national treatment sites during the countrywide ART rollout, as well as ongoing training to support the continued functioning and expansion of site efforts. In response to the MOH’s instructions, different training modules have been developed, implemented, and prioritized as emerging needs were identified (Fig. **2**).

**Fig. (3):**
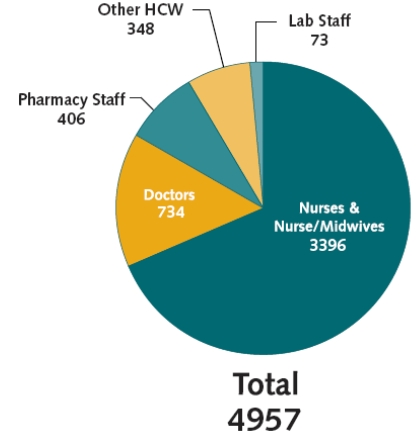
AIDS clinical care fundamentals: Cumulative training numbers per profession, 2001-2006.

Since its first offering in July 2001, *AIDS Clinical Care Fundamentals *has served as a gateway course, preparing Botswana’s HCWs to provide quality ART specific to the Botswana setting. Between July 2001 and December 2006, 4957 participants completed this module. During that period 68.5% of trainees were from the nursing professions and nearly 15% were medical doctors (Fig. **3**).

**Fig. (4):**
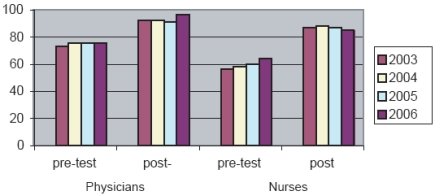
Mean pre- and post- ACCF assessement scores by profession across years 2003-2006.

Nurses and doctors participating in *ACCF* take different final examinations tailored to their clinical responsibilities. In the period 2003–2006, both professions demonstrated marked improvement in knowledge, as illustrated by the improved scores between the baseline assessments and final examinations (Fig. **4**). The mean change in pre- and post-test score was significantly higher for nurses (p<0.001) than for doctors, most likely due to lower baseline knowledge of the nurses, and doctors generally reaching maximal scores (ceiling effect) at the time of final examination.

As part of site preparation, 553 HCWs were trained in *Laboratory Fundamentals*, and 687 support staff participated in *Introduction to HIV &amp; Biosafety*. In addition, 95 HCWs completed *Integrated Practical Attachment* during the initial rollout period.

More recent additions to the KITSO curriculum, the advanced modules *Medication Adherence Counseling* and *Advanced HIV/AIDS Care and Treatment* have trained 266 and 241 HCWs, respectively. Through December 2006, 453 non-medical professionals have completed the satellite module *Introduction to AIDS Clinical Care*.

KITSO training lectures have been customized and utilized in the ART training programs of several other countries, including Tanzania [12] and Lesotho [13].

## DISCUSSION

**Fig. (5):**
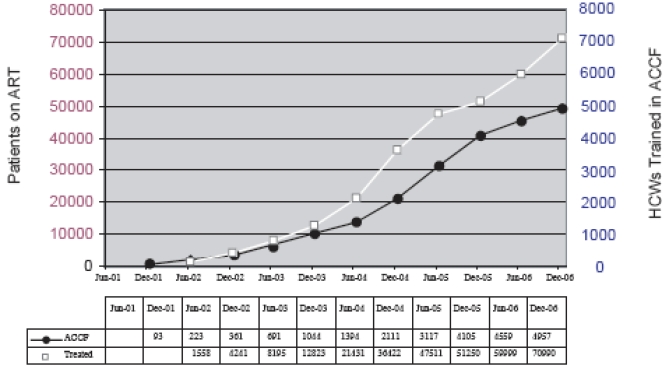
Cumulative numbers of HCWs trained in ACCF and patients on ART 2001-2006.

Training is a necessary prerequisite for the scale-up of HIV/AIDS care and treatment programs. In Botswana, there has been a demonstrated increase in both the number of HCWs completing *AIDS Clinical Care Fundamentals* and the number of patients receiving ART (Fig. **5**). With over 80,000 persons currently receiving ART in Botswana, along with thousands more in HIV care, the KITSO AIDS Training Program has been a significant factor in the successful expansion of both HIV care and ART nationally.

Standardized training modules and the coordination of KITSO training with MOH scale-up plans have been instrumental in ensuring the steady pace of Botswana’s ART rollout. The establishment of a single, standardized training curriculum ensured the consistent and uniform teaching of fundamental aspects of ART such as eligibility criteria, first-line regimens, monitoring of efficacy and tolerability, and adherence and has prepared Botswana’s health care professionals to provide high quality HIV/AIDS care and treatment. Standardized training modules and course materials also facilitated the utilization of experienced international practitioners, as well as national clinicians, to conduct *AIDS Clinical Care Fundamentals* courses and thereby enhanced both the training capacity and the quality of training.

Flexibility in implementation of training has been an important factor in achieving a high volume of training without the disruption of overall healthcare services. Training staff from large urban and semi-urban healthcare centers, as well as from small rural facilities requires different logistical approaches to training implementation. Other ART initiatives in the region will have to develop their own strategies according to their individual needs and resources [14, 15]. Operational research concerning optimal training methodologies, the effectiveness of various follow-up mechanisms, and long-term patient outcomes is certainly warranted [9].

Another important feature in the design and implementation of KITSO has been the close collaboration of national faculty with international HIV experts. This ongoing process began in 2001, and resulted in training modules that balance up-to-date medical and scientific information within the context of existing national guidelines and available diagnostic capacities. This mutual exchange of expertise has built capacity in ART and aided national clinicians in developing their own experience and expertise. When the national ART program was officially launched in January 2002, a core of capable clinicians was already in place. Training continued ahead of the rollout timetable, preparing sites in sequence for their involvement in the national program.

Efforts in sub-Saharan Africa to scale-up ART programs have been constrained by a lack of trained medical personnel characterized by both acute staff shortages and general inexperience and lack of training in ART. Recruiting additional HCWs is often slow and not always possible, thus upgrading staff training is a necessity. Innovative “task-shifting” that broadens the health care capacity base is also an option to address immediate challenges of unmet patient care needs, but this option will necessitate an even greater emphasis on standardized and expanded training efforts [3, 16].

### Ongoing Challenges

With ART now available countrywide, Botswana is well-positioned to further improve ART accessibility by extending the prescription and distribution of comprehensive ART services to existing, affiliated satellite clinics even if physicians are not available in these clinics. Some satellite clinics have already begun to provide ART treatment, and training of full staff complements in these facilities is currently underway, alongside the ongoing need for continuing education, refresher courses, advanced training, and timely information regarding revisions and amendments made to national treatment protocols.

The long-term need for ART in Botswana and elsewhere requires that adequate knowledge in HIV/AIDS care and treatment is integrated into the pre-service training curricula for all health professionals. Within the healthcare sector, the retention of trained staff with ART experience is critical to the program’s long-term success. KITSO-trained HCWs have been recruited into HIV/AIDS programs in other countries, a fact that underscores the successes of Botswana’s training and treatment efforts, but hampers expansion of the Botswana program, especially in the context of a generally understaffed health system. In addition, care should be taken to avoid transferring staff with ART experience to locations and duties that do not utilize the training they have received and the skills they have acquired.

Finally, training programs like KITSO cannot be static creations. Programs must be responsive to advances in HIV/AIDS care and to the evolving needs of the health sector they serve. KITSO seeks to be flexible and responsive as new research emerges and as Botswana’s training needs change. For example, in view of the fact that a majority of patients in the ART program are women, and in view of the revived desire to have a child following restoration of health on ART [17], a new module on *HIV and Women’s Health* is now being developed to focus on special care and treatment considerations for women living with HIV, including family planning issues.

## CONCLUSION

As a single, standardized, national training program coordinated by the MOH, the KITSO AIDS Training Program is a model of HIV/AIDS care training which can expand health care capacity in response to the HIV/AIDS epidemic. KITSO has been an indispensable element in Botswana’s nationwide rollout of ART. Through timely implementation of a standardized curriculum, broad collaboration, high quality country-specific instruction, effective monitoring and evaluation, and strong MOH leadership and coordination, KITSO could serve as a training model for many countries in the early stages of national HIV care and ART program implementation.
